# COVID-19 Vaccination Might Induce Postural Orthostatic Tachycardia Syndrome: A Case Report

**DOI:** 10.3390/vaccines10070991

**Published:** 2022-06-22

**Authors:** Melody Hermel, Megan Sweeney, Edsel Abud, Kathleen Luskin, Jose P. Criado, Robert Bonakdar, James Gray, Thomas Ahern

**Affiliations:** 1 Scripps Clinic, Department of Cardiology, 9898 Genesee Avenue, La Jolla, CA 92037, USA; hermel.melody@scrippshealth.org (M.H.); bonakdar.robert@scrippshealth.org (R.B.); gray.james@scrippshealth.org (J.G.); ahern.thomas@scrippshealth.org (T.A.); 2 Scripps Center for Integrative Medicine, 10820 N. Torrey Pines Road, La Jolla, CA 92037, USA; 3 Scripps Clinic, Department of Allergy & Immunology, 3811 Valley Centre Drive, San Diego, CA 92130, USA; abud.edsel@scrippshealth.org (E.A.); luskin.kathleen@scrippshealth.org (K.L.); 4 Scripps Clinic, Department of Neurology, 9898 Genesee Avenue, La Jolla, CA 92037, USA; criadodelvalle.jose@scrippshealth.org

**Keywords:** postural orthostatic tachycardia syndrome, COVID-19 vaccine, autoimmunity, post-vaccination, dysautonomia, inflammation

## Abstract

We report a case of new-onset postural orthostatic tachycardia syndrome in a healthy 46-year-old female after a single dose of the BNT162b2 (Pfizer-BioNTech) SARS-CoV-2 vaccine. There have been three prior reports of new-onset postural orthostatic tachycardia syndrome after COVID-19 vaccination. Predominant symptoms noted included fatigue, brain fog, headache, sinus tachycardia, and dizziness. Management includes noninvasive therapies, behavioral approaches, and pharmacologic regimens. Here, the patient presented with fatigue, palpitations, dizziness, and presyncope, with symptoms beginning 7 days after vaccination. Presenting vitals included temperature within normal limits, inappropriate tachycardia, up to 120 beats per minute, blood pressure of 128/87 mm of mercury, and 100% saturation in room air. Her management included lifestyle changes, dietary supplements, and ivabradine. Further studies are needed to evaluate prevalence, etiology, and optimal management.

## 1. Introduction

Recently, there have been reports of autonomic nervous system dysfunction, including inappropriate sinus tachycardia, orthostatic intolerance, and neurocardiogenic syncope, in patients after COVID-19 infection and vaccination. This heterogenous assembly includes postural orthostatic tachycardia syndrome (POTS), a multifactorial and often debilitating type of autonomic dysfunction that presents most frequently in young women [1]. The pathophysiology of POTS remains highly debated and may include either overactivity or failure of the sympathetic and/or parasympathetic nervous systems, resulting in overall dysregulation and subsequent disruption in homeostasis. POTS is defined as the presence of chronic symptoms of orthostatic intolerance (≥6 months) accompanied by an increased heart rate (HR) ≥30 bpm within 10 min of standing or head-up tilt (HUT) and in the absence of orthostatic hypotension (a decrease in systolic blood pressure (BP) of 20 or more mmHg and/or decrease in diastolic BP of 10 or more mmHg with upright positional change) [2,3].

Persistent autonomic dysfunction that may be induced by severe acute respiratory syndrome coronavirus 2 (SARS-CoV-2) after COVID-19 infection has been reported and recognized, so-called “Long-COVID” [4,5,6,7,8,9]. Long-COVID symptoms include, but are not limited to, tachycardia, dizziness, widespread pain, profound fatigue, migraine, brain fog, tremors, behavioral and sleep disturbances, reduced exercise tolerance, paresthesia, and exertional dyspnea [10,11]. While case reports describing new-onset autonomic dysfunction after COVID-19 infection have been increasing since the start of the COVID-19 pandemic in March 2020, there remains minimal information about adverse reactions to the COVID-19 mRNA vaccination [8,9,12,13,14,15]. 

Three reviewers systematically searched four databases (Pubmed, Google Scholar, Embase, and medRxiv) for full articles published through May 2022, using the key terms “(postural orthostatic tachycardia syndrome OR postural tachycardia syndrome OR POTS OR dysautonomia OR orthostatic hypotension) AND (COVID-19 vaccine OR COVID-19 vaccination OR SARS-CoV-2 vaccines OR 2019 Novel Coronavirus Vaccine)”. Duplicates and abstracts without full text articles were removed from the original 42 articles that returned, yielding 28 remaining articles. We identified three relevant articles that described cases of patients diagnosed with new-onset POTS after receiving COVID-19 vaccines [16,17,18]. While we cannot imply causality, here, we report a case of new-onset POTS following initial administration of the COVID-19 vaccine. In addition, we compare the previously reported post-COVID-19-vaccine POTS cases with our case. We also consider how these cases compare to the new-onset POTS cases that have been reported to develop after COVID-19 infection versus COVID-19 vaccination.

## 2. Case Presentation

In October 2021, a 46-year-old female with prior medical history of allergic rhinitis and history of COVID-19 infection presented to our outpatient integrative medicine clinic with lightheadedness, tremors, rapid heart rate, Raynaud’s Phenomenon, and marked fatigue, four weeks after receiving her first dose of the BNT162b2 (Pfizer-BioNTech) mRNA SARS-CoV-2 vaccine. Previously, in December 2020, the patient had tested positive for COVID-19, confirmed via reverse transcriptase polymerase chain reaction (RT-PCR). The patient had not yet been vaccinated when she was diagnosed with COVID-19, and she reported mild, flu-like symptoms. Within a week after her COVID-19 diagnosis in December 2020, the patient returned to her normal baseline without any new or persistent sequelae.

In September 2021, 9 months after she fully recovered from her SARS-CoV-2 viral infection, the patient was then vaccinated against COVID-19. Approximately 1.5 h after receiving her first COVID-19 vaccination on 5 September 2021, the patient developed a localized reaction on her right arm, which included erythema and urticaria. On 7 September, two days after the vaccination, she recalled dysesthesia, paresthesia, numbness, and soreness throughout the right arm, and a feeling of a fast heartbeat that she described as “tremors throughout [her] body”. On day 7 post-vaccination, the patient continued to experience burning and itching at the injection site, subjective temperature changes throughout her right arm, tremors in the right hand, nocturnal cramping in both feet, lightheadedness, and chest discomfort. She reported feeling “off” and had never previously experienced a similar array of symptoms.

Noticing her inappropriate tachycardia that persisted at rest—up to 120 beats per minute (bpm)—the patient presented to an urgent care outpatient facility, where she was found to be afebrile with a pulse of 91 bpm, a blood pressure of 128/87 mm of mercury (mmHg), and 100% saturation in room air. An ultrasound of her right arm showed normal arterial flow and no evidence of deep venous thrombosis. The patient was prescribed 10 mg oral dexamethasone and advised to take over-the-counter diphenhydramine at night as needed. On day 10 post-vaccination, the patient presented to the emergency department with worsening symptoms and was administered 10 mg intravenous dexamethasone. She was afebrile with a pulse of 107 bpm, a blood pressure of 127/84 mmHg, and 100% saturation on room air.

**Figure 1:**
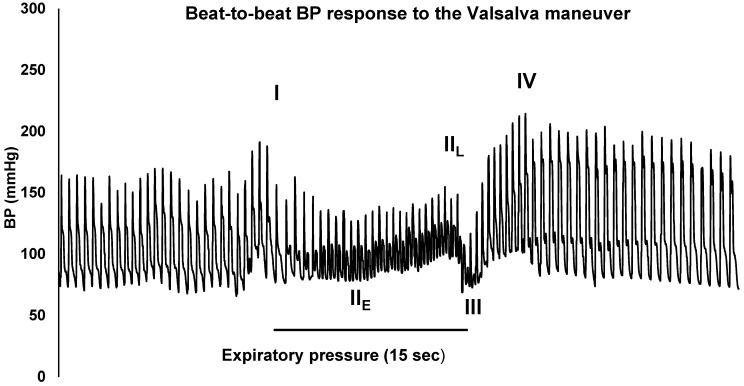
Beat-to-beat blood pressure (BP) response during the Valsalva maneuver. The VM consists of four main phases: phase I (I), early phase II (II_E_), late phase II (II_L_), phase III, and phase IV.

**Figure 2:**
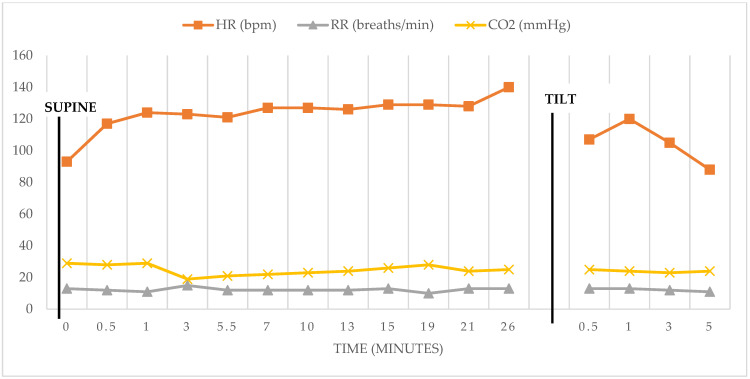
Cardiorespiratory responses from supine to head-up tilt.

On day 14 post-vaccination, the patient saw her primary care physician due to persistent symptoms, including exercise intolerance, profound fatigue, dizziness, and paresthesia. She had an electrocardiogram performed, which demonstrated sinus tachycardia with a ventricular rate of 121 bpm. Complete blood count, thyroid-stimulating hormone, total T3, and free T4 were within normal limits. Her Vitamin B12, erythrocyte sedimentation rate, C-reactive protein, chronic urticaria index (CU index), and complete metabolic panel were normal except for her aspartate aminotransferase (AST), which was elevated at 112 units/L, which was attributed to generalized muscle inflammation. The patient’s Primary Care Physician referred her to Cardiology, Allergy & Immunology, and Neurology. Neurologic testing results demonstrated normal electromyography. Allergy/immunology evaluation raised concern for localized reaction around the injection site and possible contact dermatitis related to an adhesive bandage, though upon further review with the patient, she denied any use of any bandage or dressing being applied at the time of her initial vaccine. Her persistent palpitations were not consistent with allergic etiology. Cardiology identified tachycardia in response to standing and ordered a cardiac monitor, which demonstrated episodes of sinus tachycardia with maximum heart rates of 170 bpm. Subsequent Autonomic Nervous System Testing, which included cardiovagal response to deep breathing and Valsalva maneuver assessment during tilt-table testing, was performed by the autonomic laboratory clinical manager within the outpatient center for autonomic dysfunction. The patient’s heart rate responses with deep breathing and Valsalva were unremarkable and within normal limits. Her beat-to-beat blood pressure response to the Valsalva maneuver demonstrated an excessive phase 4 overshoot (see Figure 1). During tilt-table testing, on supine to upright positioning, her heart rate increased from 93 bpm to 140 bpm maximum (see Figure 2 and Table 1). Orthostatic hypotension was not identified. The autonomic testing and tilt table maneuver reproduced the same symptoms the patient had reported at home and the cutaneous changes consistent with Raynaud’s Phenomenon, which were witnessed by the autonomic laboratory clinicians. Her end tidal carbon dioxide (CO_2_) declined significantly to 29 mmHg while the patient was in supine position and further decreased by 34% down to 19 mmHg upon head-up tilt. This reduction in her end tidal CO_2_ persisted throughout the duration of the autonomic testing procedures. 

There were no signs of deep venous thrombosis and no evidence of joint hypermobility. The patient was diagnosed with POTS and started on conservative therapy with increased salt consumption, fluid intake, and 20 mmHG compression stockings. Given her persistent tachycardia, she was started on ivabradine 5 mg BID with significant improvement in her tachycardia. She was also recommended to incorporate dietary supplements to support autonomic balance, minimize fatigue, and reduce pain and neuropathy. Our integrative medicine clinical team recommended a series of dietary supplements based on the best available evidence in an effort to reduce burdensome symptoms [19]. In February 2022, the patient began a daily regimen of dietary supplements based on these recommendations, comprising: 2000 mg Turmeric; 200 mg Vitamin C; 2000 IU Vitamin D3; 400 mg Vitamin B2; 1200 mg Enzogenol (pine bark extract); 800 mg Resveratrol Supreme with Quercetin; 1200 mg Boswellia; and 4800 mg Aged Garlic Extract Reserve Cardiovascular. At her last follow-up visit in June 2022, the patient reported improvements in her brain fog, tachycardia, dizziness, and headaches. However, she still noted persistent symptoms of Raynaud’s Phenomenon, including numbness, temperature changes, and color changes in the bilateral hands and feet.

## 3. Discussion

Here, we present a case of post-COVID-19-vaccine-associated POTS. POTS, first described in 1993, was initially classified as a post-viral illness starting after viral upper respiratory tract infections [20]. POTS has been reported post vaccination, such as for human papillomavirus vaccination and COVID-19 vaccination [21,22,23].

The patient in our report experienced POTS symptoms beginning 2–7 days after vaccination. In comparison, Carroll and colleagues recently reported a case of new-onset POTS that began 6 h after receiving the first COVID-19 vaccine [17]. Similar to the patient we have presented, Galougahi and colleagues reported a case of POTS with new-onset symptoms beginning 4 days post vaccination [18]. Likewise, Reddy et al., reported a case of post-COVID-19-vaccine-associated POTS beginning 6 days after vaccination [16]. In our reported case, the patient’s symptoms began within a similar time frame as the aforementioned cases (see Table 2). 

In our reported case, diagnostics included comprehensive laboratory evaluation of cell counts, end organ dysfunction, and comprehensive autonomic nervous system testing. Our patient’s tilt-table testing results demonstrated a heart rate increase of 47 bpm with sustained tachycardia for 26 min in the absence of orthostatic hypotension, consistent with POTS diagnosis (see Figure 2). Additionally, our patient’s beat-to-beat blood pressure response to the Valsalva maneuver demonstrated excessive phase 4 overshoot, highly sensitive for and consistent with a diagnosis of POTS (see Figure 1). Carroll’s case report included similar laboratory evaluation with detected vitamin deficiencies and abnormal tilt-table testing diagnostic of POTS, although absolute heart rate and blood pressure recordings were not reported. Galougahi’s laboratory evaluation was comprehensive and included additional autoimmune and neurologic studies, which were unrevealing, and a 5 min orthostatic tolerance test demonstrated >30 bpm increase in heart rate without concomitant hypotension. Reddy et. al. had a similar diagnostic approach. Of note, orthostatic tolerance testing was not reported, although echocardiogram demonstrated sinus tachycardia. Our case followed a similar diagnostic approach to the abovementioned cases, with the addition of more comprehensive autonomic nervous system evaluations, including cardiovagal response to deep breathing and Valsalva maneuver assessment during tilt-table testing.

Additionally, we analyzed end tidal CO_2_ as part of the patient’s comprehensive autonomic nervous system function testing. Cerebral hypoperfusion secondary to hypocapnia is well documented in the literature to be associated with POTS diagnosis; however, it has not previously been evaluated in the other post-vaccine POTS cases. Our patient’s 34% reduction in end tidal CO_2_ is also consistent with POTS (see Figure 2). Mechanistically, it has been proposed that hypocapnia leads to cerebral vasoconstriction and resultant reduced cerebral blood flow. This is further hypothesized to result in ischemic hypoxia of the carotid body, leading to chemoreflex activation, hypocapnic hyperpnea, sympathetic activation, increased heart rate and blood pressure, and peripheral vasodilation in POTS. Notably, a large retrospective study at Brigham and Women’s Hospital noted lower supine baseline end tidal CO_2_ in patients post-COVID-19 infection [13,24].

Current guidelines from The American Autonomic Society for the management of new-onset POTS in patients post-COVID-19 infection recommend conservative strategies as first-line therapies and medications, such as negative chronotropic agents as second-line therapies [25]. Ongoing research is also evaluating anti-inflammatory dietary supplements for the management of COVID-19 infection [19]. Consistent with Reddy and Carroll’s prior reports of post-COVID-19-vaccine-associated POTS, our patient was initially managed conservatively with fluids, salt, and compression stockings [16,17]. The patient in our report also benefited significantly from ivabradine and anti-inflammatory supplements. Similarly, the patients in Carroll’s reports benefited from supplementation with Vitamins D and B12 [17].

In addition to her POTS diagnosis, the patient in our case also noted symptoms of Raynaud’s phenomenon. Raynaud’s symptoms have been noted in POTS [26]. Multiple reports have associated dermatological manifestations, including Raynaud’s phenomenon, with COVID-19 infection [27,28,29]. Moreover, post-COVID-19-vaccine-associated Raynaud’s phenomenon has been reported [30]. Notably, the patient in Galougahi’s report, noted above, also experienced intermittent skin color changes with blood pooling and mottling, especially with prolonged standing, similar to the symptoms experienced by the patient in our report [18].

Of note, we are not sure what pathophysiological role our patient’s prior COVID-19 infection played in her presentation, for instance, if it could have “primed” her memory immune response, leading to a quicker response on subsequent exposure, lowered the threshold for her to develop symptoms, or created subclinical chronic inflammation. Other mechanisms that have been considered include autoantibodies, centrally mediated inflammation, or persistence of viral particles, though these remain to be confirmed. For instance, antibody-dependent enhancement by way of formation of antibody–antigen immune complexes, which trigger excessive activation of the immune cascade, has been established for other viruses, but the importance of this in SARSCoV2 infection is unknown and relevance to vaccination is thought to be low [31]. Others have also noted the need for further research of post-vaccine side effects, specifically in novel mRNA vaccines [16].

Post-COVID-19-virus-associated POTS has been reported and seems to be far more prevalent than post-COVID-19-vaccine-associated POTS [8,9,15,25,32,33,34]. Despite the handful of cases describing new-onset POTS after the COVID-19 vaccine, the vaccine has demonstrated significant protection against SARS-CoV-2 infection and reduces risk of developing Long-COVID or post-infectious POTS [35]. Therefore, the authors of this report strongly espouse the COVID-19 vaccine and strongly recommend its appropriate use according to current guidelines.

## 4. Conclusions

While there have been a few prior reports of POTS possibly linked to the COVID-19 vaccine, our case of new-onset POTS in a 46-year-old female—who experienced initial symptoms just days after the COVID-19 vaccine—contributes to the growing literature, building a greater understanding and awareness of the different presentations, diagnostics, and management strategies of this condition. While we cannot imply causality, the initial onset of POTS symptoms was closely linked in time to administration of the COVID-19 vaccination, similar to previously reported cases of POTS after COVID-19 vaccination. Future robust studies are needed to understand this potential association between new-onset POTS and COVID-19 vaccination.

## Figures and Tables

**Table 1:** Blood pressure and heart rate response from supine to head-up tilt.

Heart Rate Responses																
Test			Parameter				Result				Normal Values			Percentile %		
Deep Breathing			HR Range (bpm)				20.4				[>10.0]			46		
Valsalva Maneuver			Valsalva Ratio				1.69				[>1.51]			19		
Blood Pressure and Heart Rate Response to Head-up Tilt.																
Values with patient to 70 degrees head-up tilt																
	Supine	30 s	1 min	3 min	5.5 min	7 min	10 min	13 min	15 min	19 min	21 min	26 min	30 s post tilt	1 min post tilt	3 min post tilt	5 min post tilt
BP (mmHg)	102/69	115/75	122/85	106/77	101/76	114/80	113/83	113/77	111/82	114/83	117/84	113/78	102/68	99/65	109/72	108/74
HR (bpm)	93	117	124	123	121	127	127	126	129	129	128	140	107	120	105	88
Respiration Rate (breaths/min)	13	12	11	15	12	12	12	12	13	10	13	13	13	13	12	11
EtCO2 (mmHg)	29	28	29	19	21	22	23	24	26	28	24	25	25	24	23	24

**Table 2:** Case reports and series of POTS after COVID-19 immunization.

Author	Patient’s History	Vaccine	Presenting Symptoms/Timing	Diagnostic Test Results	Management
Our Case	46-year-old woman, history of allergic rhinitis	First Pfizer-BioNTech COVID-19 Vaccine	Hives, urticaria, fatigue onset 1.5 h after vaccination; Dizziness, tremors, numbness, brain fog, headache onset 7-days after vaccination	CBC, Inflammatory Markers, Endocrine markers, electrolytes within normal limits. LFTs normal aside from elevated AST. Autonomic Testing including cardiovagal response to deep breathing and Valsalva maneuver assessment during tilt-table testing	Fluids, salt, compression stockings 20 mmHg, ivabradine 5 mg BID, natural supplements
Carroll, 2022 [17]	30-year-old healthy female	First Oxford-AstraZeneca ChAdOx1nCoV-19 vaccination	Flu-like symptoms, dizziness, nausea, weakness, bradykinesia, brain fog, fatigue, appetite changes, loss of taste, polydipsia, numbness, tinnitus, micrographia and headaches onset 6-h post-vaccination	CBC, LFTs, Inflammatory Markers, Endocrine markers, electrolytes within normal limits. Deficiency of Vitamins B12, D2, D3, and total D. Chronically elevated D-dimer. Abnormal tilt-table test consistent with POTS, significant systolic blood pressure elevation	Replacement therapy of Vitamins B12 (1 mg intramuscular hydroxocobalamin, 3×/week) and Vitamin D (50,000 IU Stexerol-D3 weekly)
Galougahi, 2021 [18]	29-year-old healthy male	First Oxford-AstraZeneca ChAdOx1nCoV-19 vaccination	Extremeity parasthesias 4-days post-vaccination; neuralgias, tachycardia, skin-color changes with mottling and blood pooling/dependent acrocyanosis, palpitations, dizziness 2-months post-vaccination	CBC, CMP, inflammatory markers, thyroid function, and folate were normal. B12 was mildly low. Positive low-titer ANA. Electrocardiogram and echocardiography were normal. Advanced neurologic serologies and imaging were negative. 5-min orthostatic tolerance testing	Vitamin B12 injections and Amitriptyline for neuralgia, 5-weeks of steroids, and lifestyle modifications
Reddy, 2021 [16]	42-year-old male with history of hypothyroidism and B12 deficiency	First Pfizer-BioNTech COVID-19 Vaccine	Sinus tachycardia, dizziness, headaches, and fatigue onset 6-days after vaccination	CBC, LFTs, Inflammatory Markers, Endocrine markers, electrolytes within normal limits. Echocardiogram normal however noted sinus tachycardia (158 bpm)	Lifestyle modifications, compression socks, and sodium

## Data Availability

Not applicable.
